# Association of early repolarization pattern with cardiovascular outcomes in middle‐aged population: A cohort study

**DOI:** 10.1002/clc.23488

**Published:** 2020-10-26

**Authors:** Yun‐Jiu Cheng, Xiao‐Xiao Zhao, Shun‐Ping Pan, Jia‐Min Pan, Ming Zhang, Zhu‐Yu Li

**Affiliations:** ^1^ Department of Cardiology The First Affiliated Hospital, Sun Yat‐Sen University Guangzhou China; ^2^ Key Laboratory on Assisted Circulation The First Affiliated Hospital, Sun Yat‐Sen University Guangzhou China; ^3^ Department of Radiology The First Affiliated Hospital, Sun Yat‐Sen University Guangzhou China; ^4^ Department of Ultrasonography The First Affiliated Hospital, Sun Yat‐Sen University Guangzhou China; ^5^ Department of Cardiology Beijing Anzhen Hospital, Capital Medical University Beijing China; ^6^ Division of Cardiovascular Diseases Mayo Clinic Rochester Minnesota USA; ^7^ Department of Obstetrics and Gynecology The First Affiliated Hospital, Sun Yat‐Sen University Guangzhou China

**Keywords:** early repolarization, electrocardiography, epidemiology, J‐wave, sudden cardiac death

## Abstract

**Background:**

Large cohort studies provide conflicting evidence regarding the prognostic value of early repolarization pattern (ERP) in the general population, complicated by the complex or heterogeneous definitions of ERP applied in different studies.

**Hypothesis:**

We hypothesized that ERP was associated with increased cardiovascular risk with the definition of ERP recommended by the expert consensus statements.

**Methods:**

A total of 13673 middle‐aged subjects from the prospective, population‐based Atherosclerosis Risk in Communities (ARIC) study were included in this analysis. Cox models were used to estimate the hazard ratios (HRs) adjusted for possible confounding factors. ERP was defined as ST‐segment elevation ≥0.1mV at the end of the QRS or J wave on the QRS downstroke in two or more contiguous leads.

**Results:**

Compared with those without ERP, subjects with ERP had a significantly increased risk of developing sudden cardiac death (SCD) (HR, 1.48; 95% CI, 1.08–2.04) and death from coronary heart disease (CHD) (HR, 1.45; 95% CI, 1.10–1.92) after a median follow‐up of 20.1 years. ERP was significantly predictive of SCD in females, whites, younger people, and subjects with relatively low cardiovascular risk. ERP with ST‐segment elevation appeared to indicate poor cardiovascular outcomes. ERP was associated with an absolute risk increase of 93.3 additional SCDs per 100 000 person‐years.

**Conclusions:**

Our findings suggest that ERP was an independent predictor of SCD and CHD death in the middle‐aged biracial population.

## 1 INTRODUCTION

The early repolarization pattern (ERP), manifested as elevation of the QRS‐ST junction (J‐point) affecting 1% to 13% of general population, has been traditionally considered a benign entity.^1^ Nevertheless, more recent studies have shown positive, negative, and neutral relationships between ERP and cardiovascular outcomes, including sudden cardiac death (SCD), cardiac death, and death from any cause.^2^, ^3^ Interpretation of the evidence has been complicated by the complex or heterogeneous definitions of ERP applied in different studies.

In the landmark study of Haissaguerre et al., the investigators defined ERP as the presence of a terminal QRS slurring or notching, regardless of ST‐segment elevation, and found an increased prevalence of ERP among patients with history of idiopathic ventricular fibrillation.^4^ However, other studies on ERP that included ST segment elevation as a criterion reported absence of an association between ERP and cardiovascular death.^5^ In the Atherosclerosis Risk in Communities (ARIC) study, O'Neal et al. defined ERP as J wave in any leads and reported no adverse cardiovascular outcomes associated with ERP.^6^ A major methodological issue in these studies is that the definition of ERP has not been consistent. Some researchers defined ERP as the presence of QRS notching or slurring, whereas others defined it as the presence of ST‐segment elevation.

Given that considerable confusions over the definition could hamper systematic research in this area, agreed definitions of ERP have been established more recently by American Heart Association (AHA)/American College of Cardiology (ACC) Expert consensus statements.^7^, ^8^ In spite of this, these established consensus definitions have been rarely introduced in epidemiological research of ERP thereafter. As the definitions of ERP could influence the interpretation of its prognostic significance and risk stratification greatly, there is a clear imperative to apply the agreed definitions to the studies of prognostic significance associated with ERP. Therefore, our goal was to examine the long‐term cardiovascular outcomes of ERP, consistent with the established consensus definitions, in a large middle‐aged biracial cohort of US adults in the ARIC study.

## 2 METHODS

### 2.1 Study design and population

The design of the ARIC study has been described previously.^9^ In brief, participants between 45 and 64 years of age were recruited from four communities across the United States (Washington County, Maryland; Forsyth County, North Carolina; Jackson, Mississippi; and suburban Minneapolis, Minnesota) between 1987 and 1989 to take part in a prospective study of cardiovascular disease. Participants underwent a standardized evaluation of cardiovascular risk factors and returned for four follow‐up examinations (1990‐1992, 1993‐1995, 1996‐1998, and 2011‐2013). They continued to be followed via semiannual telephone calls to ascertain study end points. The examination included a 12‐lead electrocardiogram, from which the data for ERP were derived. The study was approved by the institutional review boards at all participating institution, and all participants provided written informed consent at enrollment. A detailed account of the study rationale and procedures performed at the baseline examination has been described previously in detail.

We obtained the cohort data sets from the NIH Biologic Specimen and Data Repository Information Coordinating Center (BioLINCC).^10^, ^11^ For the present analysis, we excluded the following participants^1^: subjects for whom ECGs were missing or incomplete^2^; subjects with major ventricular conduction abnormalities (eg, complete left or right bundle branch blocks), pacemakers, Wolff‐Parkinson‐White syndrome, Brugada syndrome, QRS duration ≥120 ms (in order to remove cases of significant ventricular conduction delay)^3^; subjects with acute chest pain accompanied by ST segment elevation on ECG^4^; the few ARIC subjects with race other than black or white^5^; subjects who reported the use of class I or III antiarrhythmic drugs at baseline.

### 2.2 Electrocardiographic measurement

Digital 12‐lead ECGs were obtained at baseline as described previously.^12^ ERP was diagnosed using the definition recommended by AHA/ACC Expert consensus statements.^7^, ^8^ Briefly, ERP is present if the following criteria are met: (a) ERP with ST segment elevation: the elevation above the isoelectric baseline of the segment between the end of the QRS and the beginning of the T wave in the absence of chest pain; (b) ERP with J wave: end‐QRS notch (a low‐frequency deflection at the end of the QRS complex) or slur (an abrupt change in the slope of the last deflection) at the end of the QRS on the downslope of a prominent R‐wave; (c) ST segment elevation or J wave with J‐point ≥0.1 mV in two or more contiguous leads of the 12‐lead ECG, excluding leads V1‐V3; (4) QRS duration <120 ms.

### 2.3 Ascertainment of outcomes

The primary study end point was physician‐adjudicated SCD. The methods for ascertainment of SCD events have been described previously. In order to identify SCD, all fatal coronary heart disease (CHD) events in ARIC were reviewed by an independent panel of physicians. In the present study, SCD was defined as a sudden pulseless condition that was fatal (within 24 hours) and that was consistent with a ventricular tachyarrhythmia occurring in the absence of a known noncardiac condition as the proximate cause of the death. All deaths classified as SCD had to occur outside of the hospital or in the emergency room. The secondary endpoint was CHD death because we hypothesized that the incidence of CHD death should be increased if ERP was proarrhythmic, particularly in a cohort at high risk for atherosclerotic heart disease.

### 2.4 Statistical analysis

Baseline characteristics of participants are presented as means and SDs for continuous variables and percentages for categorical variables. Tests for differences in means were assessed using unpaired *t*‐tests for continuous variables, using *χ* ^2^ tests for independence for categorical variables. We used Cox's proportional hazards models to obtain multivariate adjusted hazard ratios (HRs) for all study outcomes for subjects with vs those without ERP, as well as test for modification of the association between ERP and death for a series of potential effect modifiers. Follow‐up was defined as the time between the baseline visit until the outcome of interest, death, loss to follow‐up, or end of follow‐up.

Potential covariates included in the initial model were: age, race, sex, hypertension, smoking status, diabetes mellitus, body mass index, total cholesterol, low‐density lipoprotein level, high‐density lipoprotein level, triglycerides, fasting blood glucose, heart rate, QTc duration, and presence of left ventricular hypertrophy on ECG. Kaplan‐Meier survival curves were plotted for different outcomes. All *P* values were two‐sided and a *P* value of <.05 was considered statistically significant. SPSS software V.19.0 was used for data analyses.

## 3 RESULTS

### 3.1 Baseline characteristics

A total of 13 673 participants were included in this study and baseline characteristics stratified by ERP are given in Table 1. In the study cohort, 45.2% were male and 76.3% were white. Individuals with ERP were more likely to have history of hypertension, diabetes mellitus, and to have left ventricular hypertrophy on electrocardiogram and those without the abnormality. They were more often male, blacks, current smokers, had a higher fasting blood glucose, and higher 10‐year risk for CHD. At baseline, mean age, body mass index, cholesterol, heart rate, and QTc duration were not systematically different between subjects with and without ERP.

**TABLE 1:** Baseline subject characteristics for overall sample and by ERP status

Subject characteristics	All subjects (N = 13 673), mean (SD) or %	No ERP (N = 12 989), mean (SD) or %	ERP (N = 684), mean (SD) or %	*P* value^a^
Age (years)	54.2 (5.7)	54.1 (5.7)	54.5 (5.9)	.09
Male sex (%)	6174 (45.2)	5809 (44.7)	365 (53.4)	<.001
White (%)	10 437 (76.3)	10 108 (77.8)	329 (48.1)	<.001
Risk factors for vascular events (%)				
Hypertension	3882 (28.4)	3624 (27.9)	258 (37.7)	<.001
Diabetes mellitus	1340 (9.8)	1218 (9.4)	122 (17.8)	<.001
Currently smoking	3378 (24.7)	3157 (24.3)	221 (32.3)	<.001
Body mass index, mean (SD)	27.6 (5.3)	27.6 (5.3)	27.7 (6.1)	.79
Laboratory values, mean (SD)				
Cholesterol, mean (SD), mmol/L				
Total cholesterol	5.6 (1.1)	5.6 (1.1)	5.6 (1.2)	.83
Low‐density lipoprotein	3.6 (1.0)	3.6 (1.0)	3.6 (1.1)	.66
High‐density lipoprotein	1.3 (0.4)	1.3 (0.4)	1.4 (0.5)	.10
Triglycerides, mean (SD), mmol/L	1.5 (1.0)	1.5 (1.0)	1.4 (0.9)	.03
Fasting blood glucose, mean (SD), mmol/L	6.0 (2.1)	6.0 (2.1)	6.5 (3.1)	<.001
Risk of CHD in 10 y (%)	7.6 (7.8)	7.6 (7.8)	8.3 (7.6)	.03
Electrocardiographic findings				
Heart rate, bpm	66.0 (10.2)	66.0 (10.1)	66.1 (11.4)	.95
QTc duration, ms	415.8 (19.9)	415.8 (19.5)	416.8 (27.9)	.24
Left ventricular hypertrophy on electrocardiogram (%)	269 (2.0)	241 (1.9)	28 (4.1)	<.001

*Note*: Values are mean (SD) unless indicated otherwise.

Abbreviations: CHD, coronary heart disease; ERP, early repolarization pattern.

^a^ Significance tests for comparisons by early repolarization pattern status based on two‐sample *t* test for continuous subject characteristics and Pearson's *χ* ^2^ test for categorical subject characteristics.

In our study population, the overall prevalence of ERP was 5.0%. Distribution of ERP was as follows: 29 (0.2%) in inferior leads, 165 (1.2%) in lateral leads, and 490 (3.6%) in both territories. An ERP with ST‐segment elevation or ERP with J wave was present in 509 (3.7%) and 183 (1.3%), respectively. Representative examples of ST‐segment elevation and J wave are shown in Figure S1.

### 3.2 Association with cardiovascular outcomes

During the median follow‐up of 20.1 years, 4263 subjects (31.2%) died; of these, 666 individuals died from CHD (4.9%) and 466 were considered to be SCD (3.4%). In subjects with ERP, a total of 85 participants died from any cause (12.4%), 57 from CHD (8.3%) and 44 from SCD (6.4%). During follow‐up, 1241 subjects experienced confirmed fatal or nonfatal myocardial infarction. Of these events, 72 were subjects with ERP.

**FIGURE 1:**
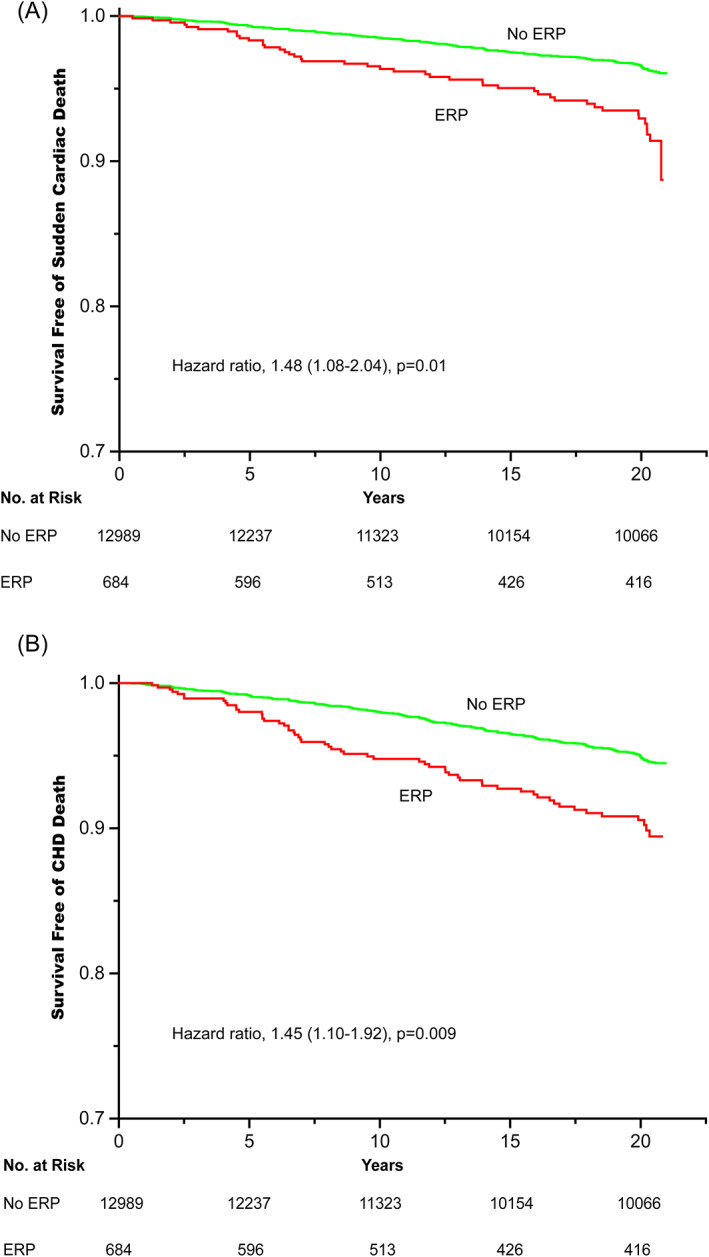
Kaplan‐Meier curves for (A) sudden cardiac death (SCD), (B) coronary heart disease (CHD) death in subjects with early repolarization pattern (ERP). Green and red lines indicate individuals without and with ERP, respectively

Table 2 shows the unadjusted and adjusted HRs of SCD, CHD death and death from any cause. In unadjusted analysis, individuals with ERP had a markedly elevated risk of SCD (HR, 2.25; 95% CI, 1.65‐3.07) and CHD death (HR, 2.02; 95% CI, 1.54‐2.65) than those without ERP. After adjustment for race, sex, and age, the point estimates seemed to be decreased, but ERP was still associated with significantly increased risk of SCD (HR, 1.55; 95% CI, 1.13‐2.13) and CHD death (HR, 1.53; 95% CI, 1.16‐2.02). Upon further adjustment for other clinical and laboratory variables in addition to demographic factors, these relationships remained significant (HR, 1.48; 95% CI, 1.08‐2.04 for SCD; HR, 1.45; 95% CI, 1.10‐1.92 for CHD death). Figure 1 shows the Kaplan‐Meier curves for SCD, CHD death in subjects with ERP. However, ERP did not seem to confer increased risk for acute myocardial infarction and death from any cause in unadjusted and adjusted analyses.

**TABLE 2:** Hazard ratios (HRs) of cardiovascular outcomes comparing subjects with ERP to those without ERP

	No ERP	ERP	*P* value
SCD (%)	422 (3.25)	44 (6.43)	
Model 1 HR (95% CI)^a^	1.00	2.25 (1.65‐3.07)	<.001
Model 2 HR (95% CI)^b^	1.00	1.55 (1.13‐2.13)	.007
Model 3 HR (95% CI)^c^	1.00	1.48 (1.08‐2.04)	.01
CHD death (%)	609 (4.69)	57 (8.33)	
Model 1 HR (95% CI)^a^	1.00	2.02 (1.54‐2.65)	<.001
Model 2 HR (95% CI)^b^	1.00	1.53 (1.16‐2.02)	.003
Model 3 HR (95% CI)^c^	1.00	1.45 (1.10‐1.92)	.009
Acute myocardial infarction (%)	1169 (9.00)	72 (10.53)	
Model 1 HR (95% CI)^a^	1.00	1.16 (0.91‐1.48)	.24
Model 2 HR (95% CI)^b^	1.00	1.05 (0.82‐1.35)	.68
Model 3 HR (95% CI)^c^	1.00	0.95 (0.74‐1.22)	.66
Death from any cause (%)	4037 (31.08)	226 (33.04)	
Model 1 HR (95% CI)^a^	1.00	1.09 (0.96‐1.25)	.19
Model 2 HR (95% CI)^b^	1.00	1.11 (0.97‐1.27)	.13
Model 3 HR (95% CI)^c^	1.00	1.11 (0.97‐1.27)	.13

Abbreviations: CHD, coronary heart disease; ERP, early repolarization pattern; SCD, sudden cardiac death.

^a^ Cox's proportional hazards model, unadjusted for any cardiovascular risk factors.

^b^ Cox's proportional hazards model, adjusted for age, sex, and race.

^c^ Cox's proportional hazards model, adjusted for age, sex, race, heart rate, QTc duration, body mass index, serum low‐density lipoprotein, diabetes, hypertension, current smoking, and presence of left ventricular hypertrophy on electrocardiogram.

Sensitivity analyses of cardiovascular outcomes associated with ERP phenotypes indicated that ERP with ST‐segment elevation was associated with elevated risk of SCD and CHD death (adjusted HR, 1.50; 95% CI, 1.06‐2.13 for SCD; HR, 1.41; 95% CI, 1.03‐1.93 for CHD death), but it was not for ERP with J wave (adjusted HR, 1.02; 95% CI, 0.51‐2.07 for SCD; HR, 1.19; 95% CI, 0.69‐2.07 for CHD death). In addition, J‐point elevation of ≥0.1 mV in any lead (including V1‐V3) did not confer increased risk for both cardiovascular outcomes after adjusting for cardiovascular risk factors, indicating that definitions of ERP could influence the results significantly.

Participants without ERP experienced an average of 169.6 (95% CI: 154.2‐184.9) cases of SCD and cases of 242.3 (95% CI: 224.0‐260.7) CHD death per 100 000 person‐years. As compared with no ERP, ERP was associated with an estimated 93.3 (95% CI: 84.8‐101.7) cases of SCD and cases of 128.4 (95% CI: 118.47‐138.2) CHD death per 100 000 person‐years.

### 3.3 Stratified analysis

**FIGURE 2:**
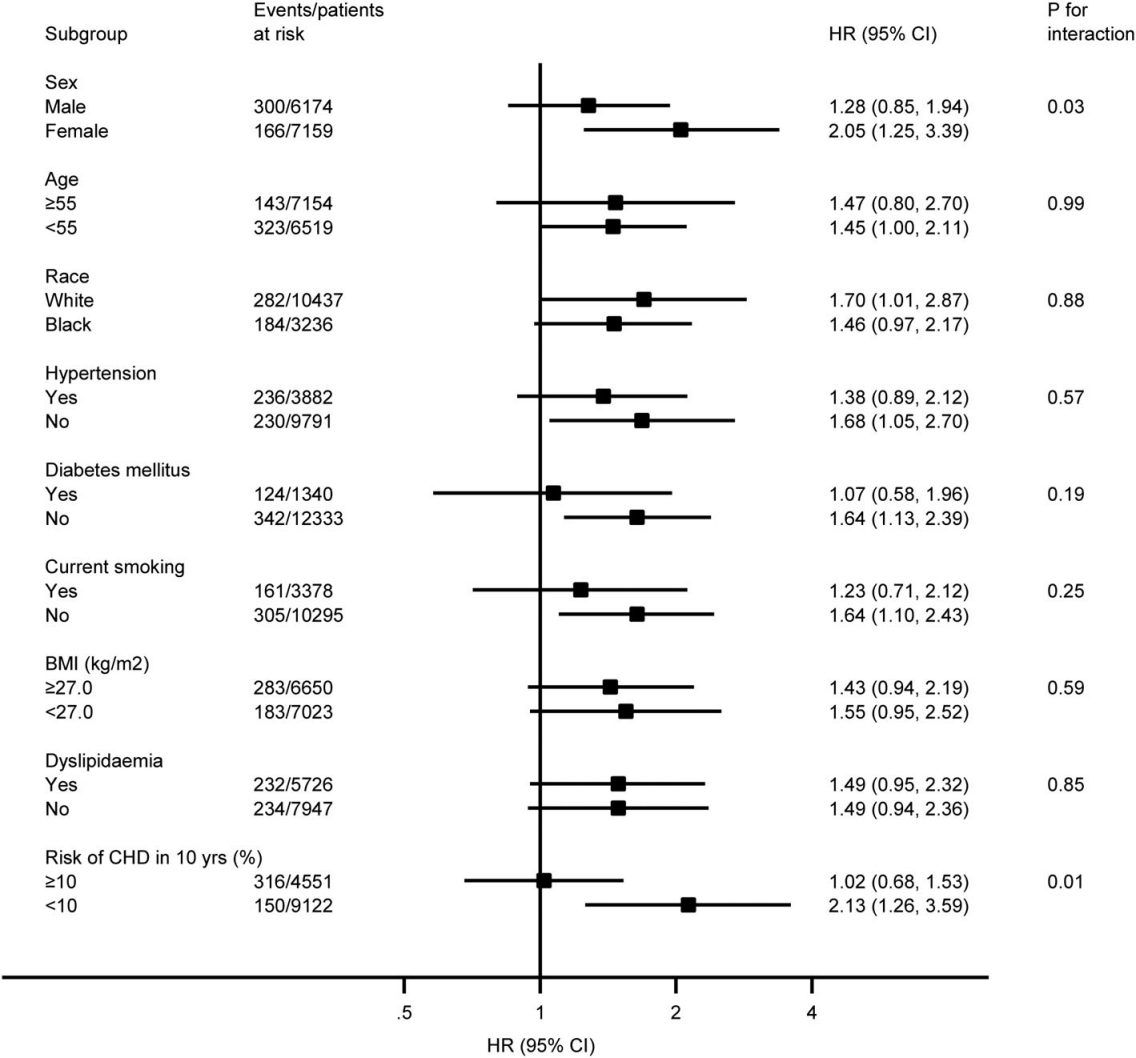
Stratified analysis of hazard ratios (HRs) of sudden cardiac death (SCD) associated with early repolarization pattern (ERP)

**FIGURE 3:**
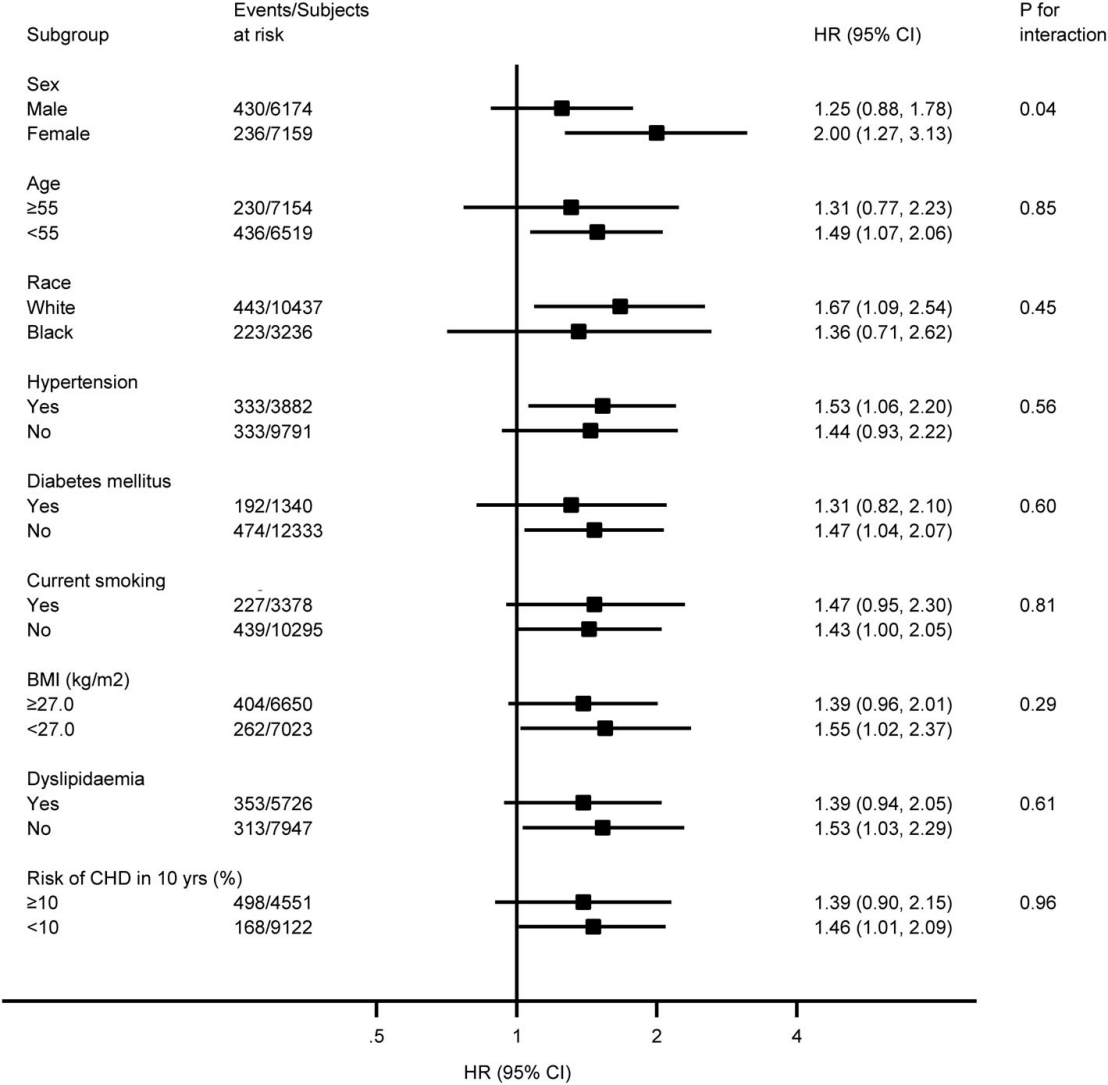
Stratified analysis of hazard ratios (HRs) of coronary heart disease (CHD) death associated with early repolarization pattern (ERP)

We performed stratified analyses across several prespecified clinical factors that might influence the outcomes. Two interaction effects between subgroup and ERP were identified: ERP was associated with a greater risk of SCD in female subjects (adjusted HR, 2.05; 95% CI, 1.25‐3.39) than male subjects (adjusted HR, 1.28; 95% CI, 0.85‐1.94; *P* = .03 for interaction), in subjects with10‐year risk for CHD <10% (adjusted HR, 2.13; 95% CI, 1.26‐3.59) compared with those with 10‐year risk for CHD ≥ 10% (adjusted HR, 1.02; 95% CI, 0.68‐3.59; *P* = .01 for interaction) (Figure 2). No significant interaction effects were identified for age, BMI, history of hypertension, diabetes mellitus, current smoking, and dyslipidemia. However, ERP was significantly predictive of SCD in those without history of hypertension, diabetes mellitus, current smoking (Figure 2). Similarly, female subjects had a higher risk of CHD death than male subjects (adjusted HR, 2.00; 95% CI, 1.27‐3.13 for females; HR, 1.25; 95% CI, 0.88‐1.78 for males; *P* = .04 for interaction). ERP was significantly predictive of CHD death in those with age <55 years, BMI <27.0, 10‐year risk for CHD <10%, and those without history of diabetes mellitus and dyslipidemia. Although risk estimates for SCD and CHD death were systematically higher in white subjects than in black subjects, the differences were not statistically significant (Figure 3).

## 4 DISCUSSION

In a large, community‐based study comprising a significant biracial population, ERP is associated with an increased risk of SCD, CHD death, even after adjustment for multiple confounding factors. The increased risk was present predominantly in females, whites, younger people, subjects without history of hypertension, diabetes mellitus, current smoking, and those with 10‐year risk for CHD <10%. ERP with ST‐segment elevation appeared to indicate poor cardiovascular outcomes. In absolute risk, ERP would account for an estimated 93.3 additional SCDs and 128.4 additional CHD deaths per 100 000 subjects per year in the general population.

The ECG term ERP has been in use by cardiologists for more than 50 years. This electrocardiographic pattern was regarded as a benign variant until 2008, when it was linked to sudden cardiac arrest due to idiopathic ventricular fibrillation by Haissaguerre and colleagues.^4^ The authors described ERP as terminal QRS slurring or notching (J wave) and did not require the presence of ST‐segment elevation in their definition. But they found that 81.8% of patients with sudden cardiac arrest had dynamic appearance of ST‐segment elevation similar to that seen with coronary spasm. However, in the case‐control study by Rosso et al., the majority of cases with cardiac arrest or sudden death were associated with J wave without ST‐segment elevation, while the ascending ST‐segment was less common in the patients resuscitated from ventricular fibrillation. In this study, notching on the R‐wave downslope, or a J wave, was considered ERP in the absence of ST‐segment elevation.^13^ Therefore, considerable confusion concerning the definition of ERP could influence its prognostic significance interpretation and clinical evaluation or treatment decisions greatly.

Findings from an analysis of the ARIC data reported in 2011 by Olson et al. did not support an association of ERP with an increased risk for SCD (adjusted HR, 1.23; 95% CI, 0.87‐1.75) or fatal/nonfatal CHD events (adjusted HR, 1.03; 95% CI, 0.89‐1.19).^14^ Similarly, a recent analysis of the ARIC data by O'Neal et al. reported no positive association of ERP with SCD (adjusted HR, 0.74; 95% CI, 0.36‐1.50) or CHD death (adjusted HR, 1.16; 95% CI, 0.87‐1.56).^6^ However, both studies did not follow the newly established consensus definitions of ERP. In the study by Olson et al., ERP was defined as a J‐point amplitude of ≥0.1 mV in any lead. In other words, J‐point elevation ≥0.1 mV in a single lead or even in leads V1‐V3 of the 12‐lead ECG was diagnosed as ERP, inconsistent with the newly established definition that J‐point elevation should be recorded in two or more contiguous leads, and that J‐point elevation in V1‐V3 should be excluded to avoid confusion with ECG patterns of Brugada syndrome or right ventricular dysplasia.^7^, ^8^, ^14^ Therefore, this method could lead to differential misclassification and could bias estimates of association between ERP and cardiovascular outcomes. In addition, O'Neal et al. defined ERP as presence of J wave in any lead of the 12‐lead ECG. This might not be appropriate, as they neither include those ECGs with ST‐segment elevation and without J wave, nor exclude those with J wave recorded in a single lead and in leads V1‐V3.^6^ Thus, the reason our group, in contrast to the studies by Olson and O'Neal who have studied the prognostic value of ERP, found clinically significant increased hazards for cardiovascular outcomes, is probably because of the disparity of the methods used.

Despite the presence of ERP is increasingly recognized as a marker of risk for SCD, the underlying biological mechanisms and pathogenesis remains incompletely understood. It is possible that the ERP phenotype of J wave or ST‐segment elevation on the ECG is caused by the transmural voltage gradient from ventricular epicardium to endocardium, due to prominence of epicardial transient outward K (+) current (Ito).^15^ Augmented dispersion of repolarization leads to disproportionate shortening of the epicardial action potential and predisposes to phase 2 re‐entry, allowing premature ventricular contractions to trigger ventricular fibrillation and SCD.^16^

Previous studies have identified that ERP with J wave was associated with increased risk of SCD, and our study is the first to our knowledge to confirm ERP with ST‐segment elevation to indicate poor cardiovascular outcomes. One might argue that the differential for ST‐segment elevation is broad and includes myocardial infarction and pericarditis, which could confound the interpretation of the prognostic value for ERP. However, we have excluded those subjects with acute chest pain and ST‐segment elevation when undergoing ECG examination. In addition, although subjects with ERP have higher cardiovascular risk than those without this abnormality in our study, ERP appeared to connote higher risk of SCD and CHD death in those with lower cardiovascular risk, especially in population without hypertension, diabetes mellitus, and current smoking. Moreover, ERP was associated with an increased risk of SCD, even adjusting for multiple cardiovascular factors. These suggest ERP could serve as an independent risk factor for SCD and CHD death in middle‐aged population. Of note, ERP confers increased risk of SCD and CHD death in females and whites but not in males and blacks, although the prevalence of ERP is higher in males and blacks than in females and whites. One explanation is that, ERP pattern in males and blacks is thought to result from higher vagal tone, and it is, thus, considered exercise related and benign.^17^, ^18^ The vagaly mediated inward potassium current during the plateau phase of the action potential could cause transmural repolarization gradient and thus ERP phenotype on electrocardiography.^19^ However, high vagal tone generally protects against malignant ventricular arrhythmias, supported by the association between ERP and bradycardia, another marker of an elevated vagal tone.^20^

A major strength of our study is the use of the ARIC cohort being characterized by long follow‐up and detailed risk factor and ECG information. A further strength of the study is the process used for ascertainment of the cause of death, which was classified using a strict adjudication process, providing a more accurate definition of SCD than is often available in epidemiological studies. In addition, application of an entirely automated method to detect this component of ERP that produces highly repeatable measurements could greatly reduce the inter‐reader variability and classification bias. However, several limitations merit discussion. First, of the group with ERP, there were only 29 or 0.2% with ERP in the inferior leads only, and 165 or 1.2% in the lateral leads only, and thus the conclusions about the prognosis of lead groupings are not able to be drawn. Second, this study included only individuals between the ages of 45 and 64 years, and thus we cannot comment on the influence of ERP outside this age range. Third, although we controlled for a number of key covariates associated with SCD and CHD death, we acknowledge that residual confounding remains a possibility.

In conclusion, this study has shown that the presence of ERP increased the risk of SCD, CHD death in middle‐aged population. ERP with ST segment elevation appears to connote a higher risk for SCD. In particular, much attention should be paid to females, whites, younger people, and subjects with lower cardiovascular risk. Future clinical and experiment studies should focus on populations with ERP at high risk, and on understanding the exact mechanisms for the malignant arrhythmias associated with ERP.

## CONFLICT OF INTEREST

The authors declare no potential conflict of interest.

## Supporting information


**Figure S1** Representative examples of ERP with ST‐segment elevation and ERP with J wave. (A) Representative ECG from a healthy female subject with no ERP. (B) Example of malignant ERP with ST segment elevation in a subject presenting with sudden cardiac death (SCD). (C) Example of benign ERP with J wave pattern in a healthy subject.

## Data Availability

The data that support the findings of this study are available from the corresponding author upon reasonable request.
