# Supplementary material for: Association of early repolarization pattern with cardiovascular outcomes in middle‐aged population: A cohort study
Source: Clin Cardiol. 2020 Oct 26;43(12):1601–8. doi: 10.1002/clc.23488 (PMC7724230; doi:10.1002/clc.23488)
Supplement: Supplementary file 1 — Figure S1 Representative examples of ERP with ST‐segment elevation and ERP with J wave. (A) Representative ECG from a healthy female subject with no ERP. (B) Example of malignant ERP with ST segment elevation in a subject presenting with sudden cardiac death (SCD). (C) Example of benign ERP with J wave pattern in a healthy subject. [file CLC-43-1601-s001.docx]

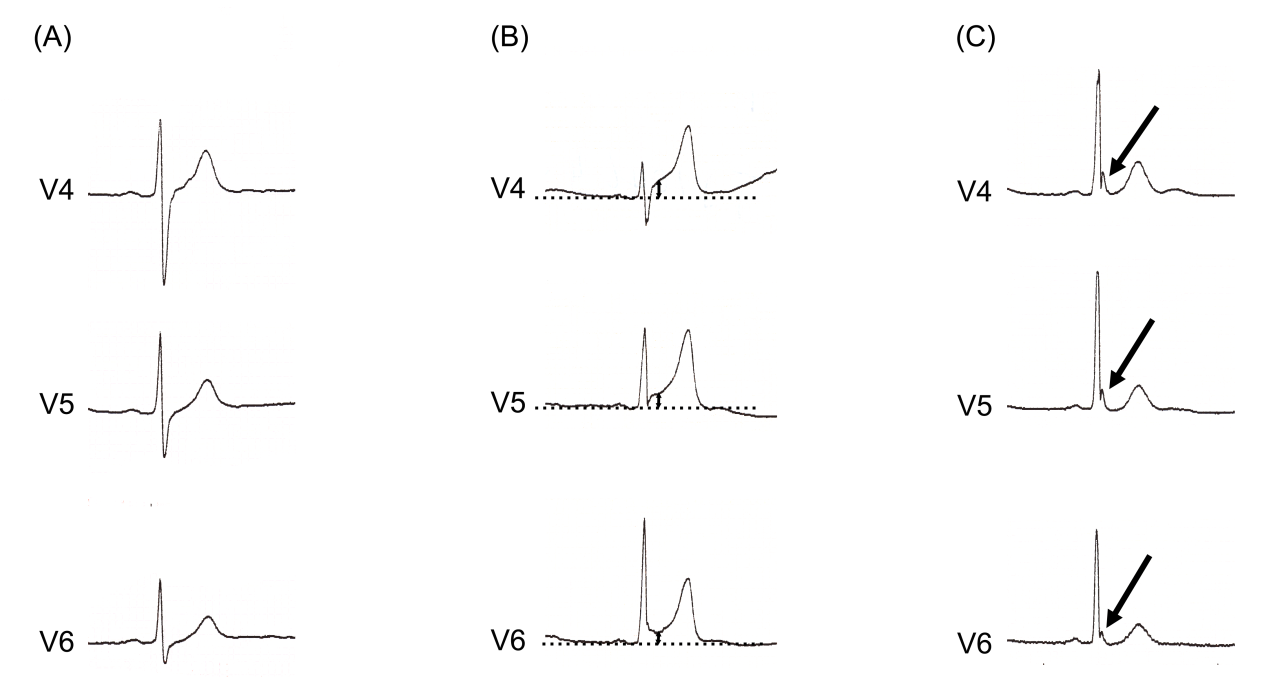


**Supplementary Figure 1.** Representative examples of ERP with ST-segment elevation and ERP with J wave. (A) Representative ECG from a healthy female subject with no ERP. (B) Example of malignant ERP with ST segment elevation in a subject presenting with sudden cardiac death (SCD). (C) Example of benign ERP with J wave pattern in a healthy subject.
